# Formative Research to Inform Market-Based Interventions to Increase Egg Purchase and Consumption in Tigray, Ethiopia

**DOI:** 10.9745/GHSP-D-21-00567

**Published:** 2022-08-30

**Authors:** Sarah McClung, Sarah Delaney, Ashley Aakesson, Kaleab Baye, Alyssa Klein, Zoe Mowl, Lydia Clemmons

**Affiliations:** a JSI Research & Training Institute, Inc., Arlington, VA, USA.; b The Manoff Group, Washington DC, USA.; c Addis Ababa University, Addis Ababa, Ethiopia.

## Abstract

Animal source foods (ASFs) have a demonstrated ability to improve child health yet are underutilized by many communities faced with malnutrition. Recognizing that improving knowledge about the benefits of consuming ASFs alone is not adequate to change behavior, the Studying Animal Food Markets in Rural Areas (SAFIRA) pilot project planned to test a market-based intervention to increasing the intake of ASFs by children 6–23 months in rural Tigray, Ethiopia. Our process of designing in-market behavior change strategies involved identifying the project’s target ASF, cocreating and testing marketing interventions, and understanding barriers and enablers driving key retailer behaviors. Qualitative research methods including focus group discussions, key informant interviews, trials of improved practices, and transect walks were used throughout 2 rounds of formative research. The first round of formative research led the project to focus on eggs, and the second round resulted in an improved understanding of the Tigrayan local food markets and egg consumption. Consumers were receptive to nutrition messaging from trusted community members and consider eggs to be healthy and affordable relative to other ASFs. Despite a willingness on the part of egg retailers in Tigrayan markets to try new practices to market eggs to consumers, formative research revealed that retailers function primarily as aggregators, moving eggs toward urban markets, correcting a foundational assumption that households routinely purchase eggs at local markets. These findings demonstrate the importance of formative research to inform design—especially in the development of context-specific behavior change interventions situated within local marketplaces.

## Abstract (precis)

We aimed to understand and address barriers and enablers related to market access, purchase, and consumption of animal source foods by children aged 6–23 months and to inform subsequent market-based interventions.

## Key Findings

- Eggs are the most likely animal source foods to be purchased for children’s consumption in Tigray, Ethiopia, and should be considered as a potential food source to achieve healthy, balanced diets for children.
- While food sellers within rural marketplaces proved willing to test innovative marketing practices to promote the sale of eggs, households do not regularly procure eggs in local markets.
- Both egg retailers and consumers indicated that households prefer to receive information about healthy food products, including eggs, from trusted community members such as religious leaders and health extension workers.

## INTRODUCTION

Malnutrition negatively impacts the health and productivity of populations around the world with nearly a quarter of all children aged younger than 5 years stunted due to chronic undernutrition.^1^ Growth faltering starts in utero and peaks when children are between ages 6–23 months when they are exposed to suboptimal diets as they transition from breastfeeding to complementary feeding.^2^ Consequently, the 2008 Lancet Nutrition series identified the first 1,000 days of life as a critical window of opportunity to prevent malnutrition and reduce stunting prevalence.^3^

Despite overall improvements in children’s nutritional status since 2000, Ethiopia continues to experience high national levels of stunting (38% in 2016), with a notably higher level in Tigray (49.2% in 2019).^4^^,^^5^ According to the 2019 Ethiopia Mini Demographic and Health Survey, only 40% of children 4–5 months were exclusively breastfed, and 23.5% of children 4–5 months were already receiving complementary foods.^6^

A year-round, safe, nutritionally adequate diet is a prerequisite of good nutritional status, especially for children aged 6–23 months (hereafter referred to as children), with consumption of animal source foods (ASFs) associated with a reduced risk of childhood stunting.^7^ Available evidence reports that, in Ethiopia, as in many other contexts, ASFs are often relatively expensive and not a part of a child’s regular diet nor the diet of other members of the household.^8^^–^^10^ For example, an evaluation implemented by the U.S. Agency for International Development project Alive and Thrive during the fasting season found that only 24% of children in Amhara consumed any ASF the previous day (18% dairy, 5% eggs, and 2% flesh foods). Furthermore, children in households where ASFs were available were 4.8 times more likely to consume them than children in households without ASFs.^8^ Within Ethiopia, ASF demand is highly seasonal due in large part to religious fasting and the frequent practice of sellers of ASFs choosing not to sell foods prohibited during fasting times, resulting in price fluctuations. Furthermore, there are constraints related to sociocultural beliefs about which foods are appropriate to feed children and a lack of caregiver knowledge of the importance of ASFs for child growth and development. Studies have demonstrated that during fasting periods caregivers are concerned about neighbors’ opinions, contamination of cooking utensils, and wastefulness of slaughtering an animal only to feed children. In 1 study, eggs and milk were viewed as more acceptable for children during fasting, but few mothers had fed them to their children during fasting periods.^8^

In Tigray, improving caregiver nutritional knowledge and specific infant and young child feeding practices has been the subject of numerous social and behavioral change interventions; however, available research suggests that improving caregivers' knowledge is not sufficient if nutritious foods are not affordable or accessible for remote, rural communities.^11^ Furthermore, evidence shows that efforts to diversify production at the farm level typically have small impacts on dietary diversity or nutrition outcomes.^12^ However, the specific interaction of barriers and enablers related to market access, purchase, and consumption of ASFs among children from the perspectives of small retailers, household food purchasers, and household caregivers are less understood.

Interventions designed around food environments—the immediate environment in which individuals make food choices for themselves and their families—may be important for sustainably improving diets. Food environments are shaped by the food systems they exist within, including economic, agricultural, industrial, health, and environmental factors that influence food availability, access, and utilization. Marketplaces can be key components of food environments and present a potential entry point for increased dietary diversity interventions because families procure an increasing proportion of their diets at local markets.^13^ The evidence supporting markets as a vital food procurement channel within food environments, when paired with the important role ASFs play in nutritious diets presents an opportunity to examine how behavior change and social marketing methods may be leveraged to promote ASFs within local markets to improve diets and nutrition. To date, little documentation exists on this topic, and much of the evidence base for behavior change interventions within the nutrition community is focused on consumers’ perspectives, with a dearth of research documenting retailers’ perspectives, particularly those of ASF retailers. Our field action report contributes to filling this gap.

Our report contributes to filling a research gap on the use of behavior change and social marketing methods to promote animal source foods at markets in order to improve diet and nutrition.

## STUDYING ANIMAL FOOD MARKETS IN RURAL AREAS PROJECT

The Studying Animal Food Markets in Rural Areas (SAFIRA) project was designed to answer the research question: can market-centered interventions successfully increase the intake of animal source foods (ASFs) in children in Tigray, Ethiopia? Project researchers hypothesized that program activities within local marketplaces could sustainably improve both supply and demand-side behaviors of retailers and consumers in a cost-effective manner to support the consumption of ASFs. The project was conceptualized before the coronavirus disease (COVID-19) pandemic and the current conflict in Ethiopia.

Two rounds of formative research were conducted in June and August 2019 (rounds 1 and 2, respectively) within rural Tigray in the Central, Eastern, and Southern zones. These zones were selected due to their relatively high population density, which made their local food markets more likely to have similar price patterns as opposed to other zones within Tigray. The study centered on both the local food markets and the localities served, as defined by households living within a 10-kilometer radius (or a 3-hour walking distance) from the market. The emergence of the COVID-19 pandemic and the conflict in Ethiopia prevented a planned third round of formative research, which was intended to inform the design and implementation of a small package of market-based interventions and a cluster randomized control trial to observe whether markets may be an effective location to promote ASFs for consumption by children.

## METHODS

To understand the factors influencing children’s consumption of ASFs, the SAFIRA project examined barriers and enablers faced by ASF retailers in Tigray in the promotion, transportation, storage, handling, and sale of ASFs to households. The project also examined consumer barriers and enablers—specifically those faced by caregivers of children—in acquiring ASFs and feeding them to their children. To identify these barriers and enablers, the project team first conducted a situational analysis and then 2 rounds of formative research in which the first round informed the second round’s design.

The SAFIRA project examined barriers and enablers faced by ASF retailers and those experienced by consumers who are caregivers of children.

Both rounds of formative research focused on 6 representative markets (3 small and 3 large), with data collection occurring outside of religious holidays and celebrations. The research team was composed of research assistants familiar with the selected communities, fluent in the local language, and experienced in qualitative research methods. Research assistants were supervised by the formative research principal investigator and research coordinator. The team collected data using locally tested and refined research tools. The findings were audio-recorded and written notes were captured in Tigrinya. The notes were translated into English and the recordings were used to clarify and add detail. Subsequently, the team conducted a thematic coding process using grounded theory.

### Situational Analysis

The situational analysis consisted of a desk review of published and gray literature as well as remote key informant interviews with project implementers, private sector professionals, policy makers, and researchers. The results of this analysis identified butchered beef, milk, and eggs as the ASFs of interest due to their common consumption in Tigrayan households.

### Ethical Approval

Before each round of formative research, the JSI Institutional Review Board (IRB) determined the study to be exempt from human subject research oversight, and the IRB of Mekelle University, Ethiopia, approved each round of research. All participants provided written or verbal informed consent for participation and for recording during focus group discussions (FGDs) and interviews.

### Formative Research Round 1

The first round of formative research systematically described aspects of the food environment, inclusive of the physical marketplace; how ASFs are sold; the demographics of retailers and con-sumers while exploring perspectives within this environment, inclusive of retailers’ perceptions, constraints, and enabling factors to building a sustainable livelihood through selling ASFs; and consumers’ household-level decision making, barriers, and enablers to purchasing and feeding ASFs to children.

During this round, research assistants conducted market transect walks using checklist guides and open-ended questions to collect information on the markets’ structure, food item availability with a focus on ASFs, social dynamics including where groups of people tended to assemble, and interactions between consumers and sellers. Then, research assistants conducted key informant interviews with 36 retailers of butchered beef, dairy products, and eggs (12 of each product) across the 6 selected markets and explored retailers’ perceptions of barriers and enablers to the sale of ASFs to households (not specifically for children), as well as their perceptions of their roles as retailers to communities in which purchasers may also be acquaintances, family, and/or friends.

Project researchers also led sex-segregated FGDs with mothers and fathers of children to understand household-level decision making. These were formed using an opportunistic sample of community health worker registers, and participating mothers and fathers were not necessarily for the same children. As the population is relatively homogenous economically, focus groups were not stratified by socioeconomic status. The FGDs included the use of a card-sorting exercise to explore perceptions of foods, including ASFs, around 5 characteristics: healthy for children, acceptable (tasty) for children, convenient to prepare, available locally, and affordable based on Hortz et al.’s research.^14^ The first 3 characteristics (healthy, acceptable, and convenient) are emotional motivators that influence parents’ decision making around what to feed their children. FGD participants initially ranked the foods into 3 categories—very, somewhat, not at all—and reached consensus on each food’s ranking. Additionally, FGDs explored family members’ roles relating to decision making about food purchasing and expenditures, and perceptions about what factors drive dietary differences across households in their communities.

### Formative Research Round 2

Based on the findings from the situational analysis, the first round of research, and the externally reported increasing levels of egg consumption by children in Tigray, project researchers identified eggs as the ASF of focus for the second round of formative research.^15^^,^^16^ Consequently, this round more deeply explored the purchasing and selling of eggs in local markets as well as interpretations of concepts promoting child egg consumption among local fathers and mothers through the use of trials of improved practices (TIPs) and concept tests.

Ten egg retailers from the selected markets were identified to participate in the TIPs, a methodology created by the Manoff Group, to test 4 improved practices over a trial period of 3 market days spanning 2 weeks.^17^ These practices were identified based on the situational analysis and first round findings (particularly findings from previous projects including Alive and Thrive and Growth through Nutrition). The 4 improved practices were discussed with the retailers and collaboratively adapted before being tested. The practices tested included (1) creating and using improved packaging to aid consumers in their transport of their purchased eggs, (2) offering a slight discount for bulk purchases of eggs, (3) selling hardboiled eggs, and (4) selling oil-coated eggs. These improved practices were identified as both low-resource and low-risk for the retailers, with the potential to increase safety, desirability, and access to eggs for consumers in local markets.

As this was a short trial focused on feasibility, the retailers absorbed the costs associated with the improved practices. After the trial period, we discussed with retailers regarding the ongoing labor and financial expenses, and research assistants interviewed the recruited retailers to determine if they tried the improved practices, modified them, planned to continue them, and observed positive or negative consequences during the trial period.

In addition to the TIPs, research assistants conducted concept testing within 4 same-sex FGDs (2 for men and 2 for women) in which each of the 30 total participants was a parent. Concepts were developed based on Round 1 findings, as well as documented experiences of other projects in Ethiopia. Each FGD centered around 6 visual prompts with an associated slogan and 1 narrative prompt; each prompt tested concepts for communicating about eggs as an affordable, healthy, tasty, and convenient food option for children and sought to measure whether parents of children were susceptible to the visual or narrative concept. The image-based concepts were developed with a local artist. Slogans translated to the local language by fluent speakers accompanied the concepts, with pronouns adjusted based on how many children were pictured.

*Love your child. Give her (them) eggs to help her (them) grow strong and smart.*

*Boiled eggs are a convenient, tasty treat. They go wherever you go!*

The slogans were read aloud to participants, so as not to assume levels of literacy. The narrative concept involved a story of a religious leader promoting feeding eggs to children during fasting, which was adapted from Alive and Thrive materials.

## RESULTS

### Round 1

#### Structured Observations in Markets

The 6 selected markets varied in consumer size from an estimated 2,000–5,000 consumers in small markets to 7,000–12,000 consumers in larger markets and were mostly located in open, unpaved areas with little permanent physical infrastructure. Only 3 of the markets had electricity, and no markets contained observable small-scale processing technologies. Within each market, retailers of similar commodities situated themselves next to one another. Butchered beef was available in 3 of the 6 markets, unprocessed milk in 1 of the 6 markets, and eggs in all of the markets. Market consumers generally congregated by sex with women near the food and men around the livestock-selling areas. Both men and women were observed talking in groups, and women were observed with children.

#### Key Informant Interviews With ASF Retailers

All 36 interviewed retailers reported selling their ASF products year-round as their main livelihood activity. Regardless of the product and size of the market, retailers reported that product demand was highly dependent on the fasting routines of the community, with periods of lowest demand during fasting periods and periods of highest demand in the days leading up to a religious celebration. Retailers typically coped with the fluctuations in demand by adjusting the price of their product.

*If there is low demand we give [the product] a low cost even with a high-cost deficit. If there is high demand we increase the price [of the product] to increase our profit.* —ASF retailer

ASF retailers’ business constraints varied across commodities. Egg retailers cited government-issued selling permits and the availability of selling locales; meat retailers cited governance-related issues and electricity to ensure cooling; and milk retailers cited the expenses of animal feed, land for the animal(s), and the availability of selling locales as the key constraints to their respective business. Despite these constraints, when asked what motivated them to continue to sell, they all mentioned the profitability of their trade.

In both small and large markets, retailers indicated that the type of customer differs for eggs, meat, and milk. Egg retailers identified their primary customers as purchasers for cafeterias and hotels while both meat and milk retailers identified individual households as their primary customers. The place of sale between retailers also differed. Most egg and meat retailers sold from their homes (7 and 9 of 12, respectively) while half of milk retailers sold their products from their homes and half sold through a seller’s association. Across ASF products, retailers reported the importance of the quality and freshness of their product, service-oriented behavior, and a dynamic of trust in the consumer-retailer relationship. Finally, ASF retailers generally did not see a need to promote their products to sell to consumers; all retailers emphasized the role of customers’ word of mouth in spreading awareness of their products throughout the community.

#### FGDs With Parents

The FGDs began with the card-sorting exercise described above to better understand ASF purchasing motivations, perceptions, and dynamics in household decision making. Both sexes reported that eggs were the most healthy and acceptable for children, convenient due to little required preparation time, and locally available. While eggs were perceived as more affordable than other ASFs, the formative research was not designed to reveal whether eggs were considered an affordable, regular addition to children’s diets in rural Tigray, and this would have been tested during project implementation. When asked about barriers to egg consumption, several participants from both groups stated that price would be a barrier for some people in the community, though they did not indicate it would be for themselves. Meat was perceived as unaffordable and unavailable with high levels of required preparation time. Furthermore, the FGDs revealed that participants from every market knew of a retailer from whom they could purchase eggs but not of re-tailers from whom they could consistently purchase meat or milk, which upheld the earlier findings from the structured observations of the markets. The average score for each targeted ASF can be found in Table 1, with possible scores ranging from a low score of 1 to a high score of 3, and each food was evaluated individually as well as in relation to each other.

**TABLE 1.** Summary of Focus Group Scores on a Card-Sorting Exercise Exploring Perceptions of Animal Source Food Products, Tigray, Ethiopia

	**Healthy for Children**	**Acceptable for Children**	**Convenient to Prepare**	**Available Locally**	**Affordable **
**Meat**	2.4	2.5	1.4	2.8	1.1
**Milk**	3.0	3.0	2.7	2.9	2.2
**Eggs**	2.9	3.0	2.7	3.0	2.8

In a card-sorting exercise with consumers, both women and men reported eggs as the most healthy and acceptable ASF for children, easy to prepare, and locally available.

The FGDs revealed that women purchased most of the household’s food and their purchasing plans change due to market price, quality, and the amount of available disposable income. When asked what they would purchase with an unlimited budget, women reported they would buy more and/or higher quality food including meat, honey, butter, and pasta while men listed non-food items such as furniture. When probed about this distinction, respondents thought the different answers could be due to different attitudes and knowledge, with women also mentioning that ill family members have different needs for food, reflecting their roles as the primary household caregivers.

When asked whom consumers purchase goods from and how purchasing decisions are made, men and women consistently reported that consumer trust in retailers was key. Respondents explained they preferred to return to the same retailer to establish credit, build confidence that they are purchasing high-quality items, and that repeated purchasing from the same retailer would make them likely to recommend that retailer to others. Respondents said that retailers from whom they frequently purchase ASFs are more likely to persuade them to try value-added ASF products. Consumers described how they had been persuaded to make a purchase by a retailer.

*“[F]or example food for children almost it is by the retailers that we became aware of the advantage of some food groups.”* —Parent

Consumers reported trust in retailers as a key factor influencing how purchasing decisions are made.

When parents were asked about barriers to obtaining more eggs, they cited price and fear of social disapproval during fasting.

### Round 2

#### TIPs

Initially, all 10 selected egg retailers agreed to try 2 of the 4 selected practices described in the methods section, with 5 sellers agreeing to try each practice (Table 2).

**TABLE 2.** Local Market Egg Retailer’s Adherence to Implementing the TIPs to Increase Consumer Egg Consumption, Tigray, Ethiopia^a^

**Practice**	**No. Retailers Who Tried TIP (N=10)**	**No. of Market Days Implemented**			**Seller Feedback**
		**3 Days**	**2 Days**	**1 Day**	
Creating and using improved packaging	4	2 sellers	2 sellers		Two sellers made new packaging devices and were somewhat interested in continuing but did not feel loss to breakage during transport and/or storage was a barrier.
Offering a slight discount to bulk purchases	4	4 sellers			Sellers were willing to try different methods but found consumers confused by discounted eggs.
Selling hardboiled eggs	6	4 sellers	2 sellers		Consumers were not interested in buying hardboiled eggs for their children.
Selling oil-coated eggs	6	5 sellers		1 seller	Sellers were interested and pleased with the results. They saw the potential of extending shelf life to sell eggs when prices were higher but also found this practice time consuming and too labor intensive.

Abbreviation: TIPs, trials of improved practices.

a There is a difference in numbers between those sellers who initially agreed to try the practice and those who actually followed through.

When negotiating the practices to try, egg retailers voiced differing perspectives on customers’ anticipated reactions to new practices. One retailer explained that retailers can promote eggs as nutritious and convenient food. Another retailer stated that communities do not always trust retailers, a sentiment that was reinforced by a retailer suggesting that trust in retailers could be increased if health extension workers promoted egg consumption. Similarly, retailers reflected different perceptions of their communities’ acceptance of novel practices.

*It is simple technology to practice and to diffuse throughout the community, because it has immediate short-term benefits. Our communities are very fast to copy new technologies if they are convinced by its benefits.* —Egg retailer

*The communities may not easily accept it. It will take some time.* —Egg retailer

During the trial, egg retailers noticed that community members were apprehensive of the new products and practices. For example, 1 retailer explained that the community did not accept hardboiled eggs on his first market day, but by the third market day, the retailer attracted interested customers. Retailers also said that hardboiled eggs are already available in markets but are seen as a social food for men and not as food for children. Despite this resistance, most retailers continued their 2 new practices over the course of all 3 market days.

As the TIPs were ongoing, some retailers slightly modified the practices while some tested more than 1 modification before settling on a strategy they intended to continue in the future. For example, 2 retailers offering a small discount to customers purchasing a high volume of eggs decided to provide 1 extra egg for those who purchase 10 or more eggs rather than advertising a discount. These retailers explained that reframing the discount as a bonus egg helped to overcome customers’ perception that a reduced price indicated a lower quality product. One retailer further built upon this practice by stating that they told customers that the extra egg was for their children thus “[raising] awareness about the importance of eggs for young children.”

During structured observations throughout the TIPs, project researchers noted that egg retailers were busy during market days, which left them little time to promote a new product. Additionally, the structured observations revealed that the retailers were predominantly engaged in aggregating activities, purchasing eggs from their suppliers (who were mostly small household farmers), and selling bulk volumes of eggs (500–3,000) to hotels or restaurants. It was rare for retailers to sell eggs to individuals, thus demonstrating that even if eggs were the most widely available ASF in the market settings, there are not many fixed points of sale to household consumers during which product promotion activities can take place. Transportation of eggs was not found to be a major issue despite a lack of sophisticated packaging technology, as retailers indicated that baskets with straw worked well and breakage was not a large concern. Similarly, retailers did not report high levels of shelf-life concerns though they appreciated the egg preservation practice and felt that lengthening shelf life could enable them to sell eggs when prices were higher.

During the TIPS, retailers described how households exercise a variety of strategies to procure eggs, including from their own production, obtaining from neighbors, or buying from someone en route to market to sell their eggs. While retailers were interested in discussing the possibility of selling more eggs to households, they were skeptical that consumers would trust them, or could afford to purchase eggs regularly. The stability of selling eggs to restaurants and hotels left the retailers with scant motivation to change their selling practices.

This research also identified the range of barriers and enablers impacting ASF retailers’ promotion, sale, and business in rural Tigray, Ethiopia, from retailers’ perspectives. The key barriers and enablers, consistent across ASF products, are captured in Table 3.

**TABLE 3.** Key Barriers and Enablers to ASF Retailers’ Promotion, Sale, and Business, Tigray, Ethiopia

**Method**	**Objective**	**Key Barriers**	**Key Enablers**
Structured market transect walk	Understand market setting and infrastructure	Lack of electricity for cold storage (to ensure food safety and increase product shelf life) Lack of permanent location to sell product Lack of promotion of product Unregulated competition Seasonal fasting	Market as social gathering place Trust between the consumer and retailer Profitability of products
Key informant interviews with ASF retailers	Understand retailers’ perceptions of constraints and enabling factors for making a profitable and sustainable livelihood from selling ASFs in local markets		
TIPs with egg retailers Market observation with retailer Practice negotiation Practice follow-up	Test feasibility of improved practices with egg retailers Observe current practice Reveal which relevant improved practice(s) that egg retailers are willing to try Reveal which relevant practices retailers are able to sustain over the trial period; adaptations that retailers adopt; perceptions of benefits and negative consequences	Lack of time to promote product due to business of market Perception among consumers that retailer is promoting new practice for purely financial gains Household consumers are not their usual buyers	Curious consumers Innovative mindset among retailers

Abbreviations: ASF, animal source foods; TIPs, trials of improved practices.

#### Consumer Concept Testing

All of the participants appreciated the narrative concept of community-based religious leaders promoting egg consumption by children, as they shared that religious leaders are usually a highly trusted source of information. Participants identified other prominent community members including godfathers, health care workers, health extension workers, and elders as effective sources for promoting egg consumption among children. Furthermore, all of the tested visual concepts that positioned eggs as a convenient, tasty, and healthy food for young children, and feeding young children eggs as an act of love, were well received regardless of the participants’ sex. There was a slight preference among both sexes for the image that showed parents feeding their children eggs together.

*The presence of both father and mother to treat their child is an indication of love. [This] matches with the concept of “love your child and give them eggs to help them grow strong and smart!* —Parent

Formative research findings indicated that interventions to reach consumers with social marketing activities promoting eggs at local markets could be an effective approach to increase demand for eggs. Consumers are open to motivational messages with clear calls to action on how to prepare and feed eggs to children, even during fasting times.

Formative research findings indicated that interventions to reach consumers with social marketing activities promoting eggs at local markets could be an effective approach to increase demand for eggs.

However, consumers indicated that health workers, religious leaders, and elders remain trusted sources of information when it comes to the health benefits of foods for their children, suggesting that new messaging would be more likely to be acted upon if they came from trusted sources instead of egg retailers.

Furthermore, although consumers would be open to messages from trusted sources explaining it is socially acceptable and desirable to feed eggs to children, even during fasting times, they want to see neighbors making this change before they would feel comfortable making it themselves. This finding is promising for other interventions aiming to increase the accessibility and demand for eggs in these areas of Tigray, as the project researchers found that households already believe that eggs are healthy, tasty, convenient, and more affordable than other ASFs.

## DISCUSSION

The SAFIRA project aimed to increase children’s consumption of ASFs, in part by increasing knowledge about and demand for eggs in local markets by consumers through social and behavior change activities. The 2 rounds of formative research were designed to inform these social marketing and behavior change interventions but due to the ongoing COVID-19 pandemic and the current insecurity in Tigray, the project was not able to continue its activities as planned.

The situational analysis and formative research findings indicated that social marketing activities carried out by trusted actors such as religious leaders or community health workers could be an effective approach to increase parents' practice of feeding eggs to children. This finding emerged from the concept testing and reinforced Alive and Thrive’s finding that suggested effective interventions should involve religious leaders and health professionals to reinforce key nutrition-related behaviors.^18^ Additionally, SAFIRA’s findings further aligned with Alive and Thrive in that mothers were willing to add additional ASFs (especially eggs and milk) into their child’s diet during the fasting season, if they were reassured that this would not break their fast and that they were confident that neighbors would not negatively judge them and were doing the same.

Our findings indicate that social marketing activities carried out by trusted actors such as religious leaders or community health workers could be an effective approach to increase parents' practice of feeding eggs to children.

Based on the findings from the Round 1 FGDs, SAFIRA identified markets as a feasible location to test nutritionally focused social marketing activities that SAFIRA had planned to conduct. Additionally, the research demonstrated that consumers are open to motivational messages with clear calls to action on how to prepare and feed eggs to children, even during fasting times. Price was another motivator for consumers, and while eggs were perceived to be the most affordable ASF, they were still considered expensive. This research was not designed to determine a price point that would make eggs more affordable.

Unexpectedly, the findings indicated that it may not be fruitful to work with egg retailers for point-of-sale promotion activities, since most of the retailers in these local markets function as aggregators, buying from small producers and collecting quantities large enough to sell to hotels and other customers in towns. Egg retailers operating in both the local markets where project researchers conducted the TIPs as well as those operating in the major city, Mekelle, spoke of the predictable demand from hotels and restaurants, even during fasting times. For some retailers, although the price per egg would be similar in both market locations, the assurance that their eggs would be sold motivated them to prioritize city markets. The motivators observed among egg retailers were largely in response to market dynamics, including demand levels by households, potential profitability levels, and doubts about how their role in sharing nutrition advice would be perceived.

## CONCLUSION

The SAFIRA project demonstrated the importance of conducting formative research to understand consumers’ and retailers’ perspectives related to sale, purchase, and consumption behaviors—all of which are integral to a project operating within complex food environments.

Moving forward, we would advise fellow researchers and practitioners in this domain to consider the complexity of the food environments they examine from the perspectives of both individuals selling product and those shopping in the market. Furthermore, the project’s experience demonstrates the importance of the role of formative research to explore assumptions before designing interventions. In these complex contexts, participatory and flexible formative research is critical. Observation, participatory FGDs, and TIPs are examples of such research methods. Market-based interventions may be cost-effective components of broader strategies to increase demand for ASFs and other nutritious foods, which SAFIRA had hoped to explore during implementation. Such interventions must be based on an in-depth understanding of the food environments in which they operate, which may vary in ways that negate foundational assumptions.

## Notes

**Peer Reviewed**

**First published online:** July 29, 2022.
